# The Relationship Between Social Media Use Purposes, Healthy Lifestyle Behaviors, and Metabolic Parameters in Adolescents With Type 1 Diabetes

**DOI:** 10.1155/pedi/2096744

**Published:** 2025-06-17

**Authors:** Hakan Avan, Nimet Barna

**Affiliations:** ^1^Nursing Department, Kahramanmaraş Sütçü İmam University, Afşin School of Health, Kahramanmaraş, Türkiye; ^2^Diabetes Nurse, Gaziantep Cengiz Gökçek Maternity and Pediatrics Hospital, Gaziantep, Türkiye

**Keywords:** adolescent, healthy lifestyle, nurse, social media, type 1 diabetes

## Abstract

**Objectives:** This study aims to determine the relationship between social media use, healthy lifestyle behaviors, and metabolic parameters in adolescents with Type 1 diabetes (T1D).

**Methods:** The study was conducted with descriptive and cross-sectional design, andwas carried out on 108 adolescents, aged 11–18, who were diagnosed with T1D mellitus (T1DM) via Google Form. An adolescent descriptive information form, the Scale of Social Media Use Purposes (SSMUP), the Social Media Addiction Scale (SMAS), and the Adolescent Lifestyle Profile (ALP) were used in the study. The data were analyzed using descriptive statistics such as number, percentage, mean and standard deviation (SD), and statistical methods such as *t*-tests and Pearson correlation analysis.

**Findings:** The results of the study indicated that social media addiction is significantly related to adolescents' interpersonal relationships and metabolic parameters, while the purposes of social media use are positively associated with physical activity, healthy eating, and interpersonal relationships.

**Conclusions:** Nurses should closely maintain vigilant oversight of social media use and interpersonal communication among adolescents with chronic diseases, create social media content appropriate for diabetes management, and organize trainings on the effects of social media use.

## 1. Introduction

Type 1 diabetes (T1D) is a chronic disease characterized by insufficient insulin production, typically occurring during childhood or adolescence. This condition can directly affect many metabolic parameters and healthy lifestyle behaviors that impact an individual's quality of life [1, 2]. Especially adolescents widely adopt social media usage and interact on these platforms in areas such as friendships and social relationships, entertainment and passing time, self-expression, acquiring information and education, visual sharing, esthetics, and so on. The impact of social media on healthy lifestyle behaviors and metabolic parameters in adolescents has become an important area of research today.

Social media, in addition to being a tool that enhances social interactions among young people, also plays a significant role in acquiring and sharing health information. It has been determined that the level of trust in social media has a meaningful and positive effect on acquiring health information in digital environments [3]. This situation indicates that social media use can have an impact on acquiring health information and healthy lifestyle behaviors. Especially, adolescents' access to health information through social media is of critical importance in diabetes management.

However, it is believed that social media use is not limited to information acquisition, but can also affect individuals' lifestyles and health behaviors. A study conducted by Hygen et al. [4] examined the relationship between digital social interactions, loneliness, and mental health issues in adolescents. The study shows that adolescents' interactions with their peers reduce mental health problems and levels of loneliness [4]. These findings suggest that social media use can positively influence healthy lifestyle behaviors by enhancing adolescents' social interactions.

The purposes of social media use in adolescents with T1D emerge as an important factor in health management. The NutriWalking app, developed by Hartzler et al. [5], is a mobile health application designed to support individuals in adopting healthy lifestyles [5]. Such applications can help adolescents track their physical activity and eating habits, thereby promoting healthy lifestyle behaviors. Therefore, it is an important topic to investigate how social media applications play a role in health management for adolescents with T1D.

Consequently, the relationship between the purposes of social media use and healthy lifestyle behaviors and metabolic parameters in adolescents with T1D is a complex issue that requires a multidisciplinary approach. Social media is considered an important tool for adolescents in acquiring health information, establishing social interactions, and developing healthy lifestyle behaviors. Therefore, increasing research in this area will allow us to better understand the relationships between social media use and diabetes management in adolescents.

Additionally, it is crucial for pediatric nurses, within their roles in protecting and promoting child health, to identify potential issues and plan nursing interventions. These nurses not only treat diseases but also take on the responsibility of protecting children's physical and mental health by promoting healthy lifestyles. In this context, identifying the relationship between the purposes of social media use, healthy lifestyle behaviors, and metabolic parameters in adolescents with T1D will shed light on diabetes management for nurses, parents, and adolescents.

## 2. Method

This research is designed as a descriptive and cross-sectional study to determine the relationship between the purposes of social media use, healthy lifestyle behaviors, and metabolic parameters in adolescents with T1D.

### 2.1. Design of the Study and Participants

**Figure 1:**
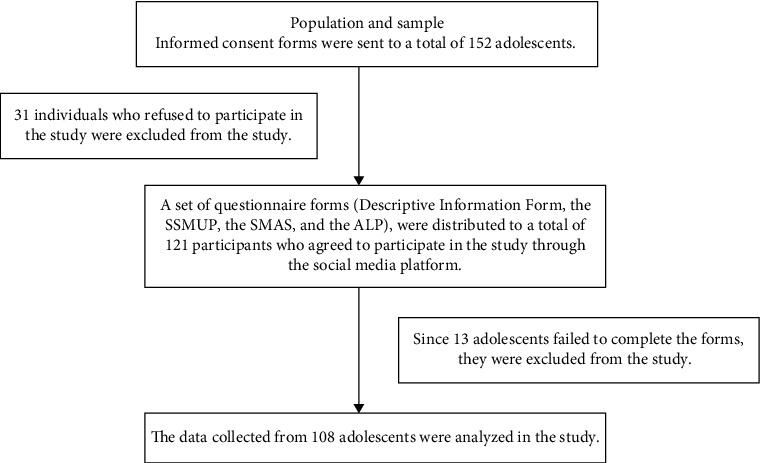
Flow chart of the study.

The study used purposive sampling method. Adolescents who were aged between 11 and 18 years, were diagnosed with T1D mellitus and were residing in Gaziantep were included in the study. Adolescents who submitted incomplete and incorrect data (13 people) were excluded from the study. The informed consent form for the research was electronically sent to the adolescents and their parents via a Google Forms link, and their consent was obtained. The researchers distributed the questionnaire and the scales, prepared on the electronic environment, to social media (WhatsApp) groups of the adolescents who agreed to participate in the study between 1 and 30 September 2023. The adolescents were asked to voluntarily complete the questionnaire forms. A post-power analysis was done to calculate the sample size. A power of 99% and an effect size of 0.4 were calculated. Based on the post hoc analysis, it was determined that the sample size (*n* = 108) was sufficient. The research flowchart is presented in Figure 1.

### 2.2. Data Collection

An adolescent descriptive information form, the Scale of Social Media Use Purposes (SSMUP), the Social Media Addiction Scale (SMAS), and the Adolescent Lifestyle Profile (ALP) were used as data collection tools in the study.

### 2.3. Adolescent Descriptive Information Form

This is a 15-item form prepared by the researchers upon literature review [6, 7, 8].

### 2.4. SSMUP

The scale developed by Solmaz et al. [9] and revised by Çömlekçi and Başol [8] is a 5-point Likert type with 11 items. The mean value of the scale is calculated, and the scale score ranges from 1 to 5. The scale has no cutoff point, and as the score rises, so does the level of social media use purposes [9, 8]. Cronbach's Alpha coefficient was 0.819 in the original version of the study; whereas, it was calculated to be 0.817 in this study.

### 2.5. SMAS

The scale, developed by Günüç [10] and revised by Çömlekçi and Başol [8], which conducted its validity–reliability study, includes seven items in 5-point Likert type. The mean value of the scale is calculated and the scale score ranges from 1 to 5. The scale has no cutoff point, and as the score rises, so does the level of social media addiction [10, 8]. While Cronbach's Alpha coefficient was 0.850 in the original version of the scale, it was calculated as 0.937 in this study.

### 2.6. ALP

Hendricks, Murdaugh, and Pender conducted the validity and reliability study of the scale in 2006. The scale makes it possible to assess the healthy lifestyle behaviors of adolescents. Ardıç and Esin [6] conducted the Turkish validity and reliability study of the scale. Its subscales are physical activity, nutrition, stress management, and interpersonal communication. The scale comprises 22 items that are rated on a 4-point Likert type. The scale has no cutoff point, and as the score rises, so does the level of positive health behavior [6]. Cronbach's Alpha coefficient was 0.84, 0.77, 0.61, and 0.68, respectively, in the original version of the scale; whereas, it was calculated to be 0.86, 0.74, 0.84, and 0.84 in this study.

In the study, scale scores and metabolic parameters (HbA1c and fasting glucose level) were the dependent variables and sociodemographic questions were the independent variables. The data were analyzed with strict adherence to confidentiality principle and no bias took place.

### 2.7. Data Analysis

The data were analyzed in the SPSS 29 software confidentially. The data were analyzed using descriptive data such as number, percentage, mean and standard deviation (SD), and statistical methods such as *t*-test, ANOVA test, and Pearson correlation analysis. Skewness and Kurtosis (±1) values were used to analyze whether the data is normally distributed; whereas the Levene's test was run to analyze the homogeneity of the variances. Cronbach's alpha test was run to calculate the internal consistency coefficient. The data were further analyzed with post hoc Tukey and Gabriel tests to identify the group from which the difference came. *p* values of ≤0.05 were considered as statistically significant.

### 2.8. Ethical Considerations

The approval was obtained from university ethics committee (Decision No. 2023-30 dated 01/08/2023) in order to conduct the study. The adolescents and parents who participated in the study were informed about the purpose and process of the study through the Google Form. The researcher obtained the authors' permission via e-mail to use the scales. No personal data was collected in the Google Form.

## 3. Findings

This section of the study will analyze the findings obtained from 108 adolescents diagnosed with T1D mellitus who were voluntary to participate in the study.

It was determined that 58.3% of the adolescents were female, 88.9% had a nuclear family, 65.7% were in their early adolescence period, 50.9% had a family income less than expenses, and 25% had problems with their parents (Table 1).

In the study it was found that 23.1% of adolescents used the information they learned from social media to treat their diabetes. The social media platforms on which they learned information were Instagram (59.3%), YouTube (38.9%), and WhatsApp (28.7%), respectively. A total of 49.1% of the adolescents spent 121 min and more on social media daily (Table 2).

Table 3 presents the range, median, mean, and SD values of the main variables.

Table 4 shows that there were significant correlations between the variables. There is a significant positive relationship between social media addiction and the purposes of social media use (*r*: 0.259; *p*  < 0.01), and a significant negative relationship with interpersonal relationships (*r*: −0.213; *p*  < 0.05). A significant positive relationship was found between the purposes of social media use and physical activity (*r*: 0.196), nutrition (*r*: 0.193), and interpersonal relationships (*r*: 0.190; *p*  < 0.05).

A significant relationship was found between social media addiction and HbA1c levels, one of the metabolic parameters of adolescents (*p*  < 0.05). It is believed that this relationship arises from adolescents with high levels of social media addiction having poor HbA1c values. A significant relationship was also found between social media addiction and adolescents' fasting blood glucose levels (*p*  < 0.05) (Table 5). This effect was considered to be due to the high fasting blood glucose levels of adolescents with high social media addiction levels.

## 4. Discussion

This study was conducted to examine the relationship between the purposes of social media use, healthy lifestyle behaviors, and metabolic parameters in adolescents with T1D.

It was observed that there was a positive and significant correlation between social media addiction and social media use purposes. This suggests that social media use purposes lay the groundwork for social media addiction. Individuals use social media platforms for many purposes, including communication with others, accessing news, browsing the internet, preparing for educational activities, seeking information related to their treatments, and engaging in leisure activities [11–13]. The time spent on social media contributes to social media addiction rather than social media use purposes [14, 15]. Prolonged time spent on social media for various reasons may lead to social media addiction in the future.

A significant negative correlation was observed between social media addiction and interpersonal relationships. Social media addiction impairs face-to-face communication skills negatively [16, 17]. Adolescents with social media addiction mostly engage in interpersonal communication activities through social media instead of communicating directly [18]. The simplification of the interpersonal communication process by social media lies among the main reasons why social networks are preferred for communication among adolescents [19]. In fact, adolescents with high levels of addiction to smartphones, social media, and online games establish more interpersonal interactions with their online friends [20]. Social media addiction in adolescents significantly and positively affects interpersonal alienation [21], as well as personality traits and family communication [22]. Adolescents having a disrupted communication with their families will be deprived of parental support in diabetes care. Less parental support would lead to disruptions in diabetes care and metabolic parameters [7]. The findings indicate that there is a significant negative relationship between social media addiction and interpersonal communication, which is a healthy lifestyle parameter, in adolescents. Adolescents may not be aware of the disruption of their interpersonal relationships due to social media addiction. They may see this process simply as a part of their daily lives. It is important for the health of adolescents that nurses closely follow-up social media use and interpersonal communication in adolescents who experience social isolation due to chronic illness and intervene early in possible deviations.

It is observed that there is a significant positive relationship between the purposes of social media use and physical activity. It facilitates self-management of behaviors related to physical activity and quality of life in adolescents who use social media to get information about exercises [23]. In fact, adolescents exhibit positive attitudes towards social media used for exercise and physical activity [24]. However, it appears that individuals who were instructed on physical activity by social media had fewer social media addictions and higher levels of physical activity [25]. Adolescents who use social media not only as a source of information but also share content related to their physical activity are more determined to engage in moderate to severe physical activities [26]. Failures in physical activity are caused by social media usage that is unrelated to it [27]. In fact, it leads to significant differences in levels of vigorous physical activity between those who are addicted to social media and those who are not [28]. Frequent, prolonged, and unconscious social media use significantly affects the physical activity levels of adolescents [27, 29].

A significant positive correlation was observed between social media use and a healthy diet. Access to information about nutrition quality on social media appears to facilitate the regulation of behaviors associated with diet quality [23]. In this context, adolescents with T1D use the internet and social media more to seek information about their disease and daily insulin adjustments [13, 30]. Unintended and prolonged use of social media not only affects adolescents' healthy dietary habits [29] but also they encourage orthorexia nervosa [31], increases in BMI *z*-score [32], and a higher risk of eating behavior disorders [31].

A significant positive correlation was observed between social media use purposes and interpersonal relationships. Adolescents use social media in interpersonal interactions. Older adolescents use social media to communicate and interact with others more than their younger counterparts. As the internet and social media use of adolescents rises, their use of social media for interpersonal interactions and their social media addiction also increases [11]. Interpersonal alienation develops in adolescents who use social media for longer times, which mediates social media addiction and learning difficulties [21]. The findings show that the purposes of social media use in adolescents are strongly associated with healthy lifestyle parameters such as physical activity, healthy eating, and interpersonal communication. Adolescents with T1D have expressed their interest in using social media as a means to promote diabetes management and improve interaction with their healthcare providers specializing in diabetes care [13, 33] Within this scope, it is considered important for pediatric nurses to create appropriate social media content for the management of diabetes in adolescents with T1D and to organize trainings on the positive and negative effects of social media use to protect and improve adolescent health.

It is observed that social media addiction has a significant relationship with HbA1c values and fasting blood glucose levels. It has been reported that adolescents change their treatment routine and cause poor glycaemic control as they spend time on internet browsing, video gaming, and social media platforms until midnight [34]. Besides spending time on social media for prolonged periods, disseminating misinformation about health also negatively affects the health conditions of adolescents [35]. It is assumed that the unconscious and aimless use of social media is negatively associated with the metabolic parameters of adolescents with T1D.

## 5. Limitations

There are some factors that impose limitations on the generalizability of the findings presented herein. First, the data were collected via Google Forms rather than face-to-face interviews. Second, we lack information regarding adolescents who refused to participate in the questionnaire. Moreover, since the study was conducted in a single province, it may not be possible to generalize the results to a wider population.

## 6. Conclusions

As a result of the research, it was found that interpersonal relationships are negatively associated with social media addiction. The purposes of social media use were positively associated with physical activity, healthy eating, and interpersonal relationships. It was also determined that social media addiction has a negative relationship with the metabolic parameters (HbA1c values and fasting blood glucose levels) of adolescents with T1D.

## 7. Relevance to Clinical Practice

Social media use purposes affect healthy lifestyles. Creating social media content suitable for managing diabetes in adolescents with T1D mellitus (T1DM) and organizing trainings on the positive and negative effects of social media use by pediatric nurses can provide adolescents with access to correct health information and achieve optimal metabolic parameters.

## 8. Patient or Public Contribution

The study suggests that adolescents with social media addiction may suffer from interpersonal communication problems. Close follow-up of social media use and interpersonal communication in adolescents who are socially isolated due to chronic illness and early intervention in possible deviations by nurses can protect and improve their health. Determining social media use in adolescents with T1D will contribute positively to adolescent and public health.

## Figures and Tables

**Table 1:** Distribution of adolescents with type 1 diabetes mellitus according to their socio-demographic characteristics (*n* = 108).

Socio-demographic characteristics	*n*	%
Gender		
Female	63	58.3
Male	45	41.7
Family type		
Nuclear family	96	88.9
Extended family	7	6.5
Broken family	5	4.6
Age		
11–14 Years (early adolescence) 15–18 Years (moderate adolescence)	71 37	65.7 34.3
Family income level		
Income less than expenses	55	50.9
Income equal to expenses	38	35.2
Income more than expenses	15	13.9
Having problems with parents		
Yes	27	25.0
No	81	75.0

**Table 2:** Social media usage characteristics of adolescents with type 1 diabetes (*n* = 108).

Social media features	*n*	%
Using information obtained from social media		
Yes	25	23.1
No	83	76.9
Social media source of information^a^		
WhatsApp	31	28.7
YouTube	42	38.9
Instagram	64	59.3
TikTok	27	25.0
Twitter	7	6.5
Facebook	7	6.5
Other	9	8.3
Time spent on social media		
60 min and under	32	29.6
61–120 min	23	21.3
121 min and more	53	49.1

^a^Percentages were analyzed among themselves.

**Table 3:** Basic statistical measures for the main variables of the study (*n* = 108).

Variables	Min	Max	Mean	SD
SMAS	1.00	5.00	2.189	1.109
SMUP	1.55	5.00	2.824	0.705
Physical activity	7.00	24.00	15.120	4.395
Diet	7.00	24.00	16.768	3.611
Stress management	6.00	20.00	14.379	3.608
Interpersonal relationships	6.00	20.00	14.527	3.674

**Table 4:** Correlation analysis results for the variables of the study.

	SMAS	SMUP	Physical activity	Nutrition	Stress management	Interpersonal relationships
SMAS SMUP Physical activity Diet Stress management Interpersonal relationships	1 0.259*⁣*^*∗∗*^ −0.062 0.020 −0.098 −0.213*⁣*^*∗*^	— 1 0.196*⁣*^*∗*^ 0.193*⁣*^*∗*^ 0.147 0.190*⁣*^*∗*^	— — 1 0.350*⁣*^*∗∗*^ 0.640*⁣*^*∗∗*^ 0.494*⁣*^*∗∗*^	— — — 1 0.464*⁣*^*∗∗*^ 0.399*⁣*^*∗∗*^	— — — — 1 0.702*⁣*^*∗∗*^	— — — — — 1

Abbreviations: SMA, Level of Social Media Addiction; SMUP, Level of Social Media Use Purposes.

*⁣* ^*∗∗*^ *p* < 0.01, *⁣*^*∗*^*p* < 0.05.

**Table 5:** Analysis results of the effect of social media addiction on metabolic parameters of adolescents with type 1 diabetes mellitus (*n* = 108).

Variables	Social media addiction		
	*n*	Mean ± SD	Test
HbA1c value			
Medium (between 6.5 and 7.4)	23	1.59 ± 0.52	***t* = 4.541** *p* ≤ 0.001
Poor (7.5 and above)	85	2.35 ± 1.17	
Fasting blood glucose level			
Between 70 and 130 mg (dl)	30	1.88 ± 0.90	***t* = 2.018** *p*=0.048
131 mg (dl) and above	78	2.30 ± 1.16	

Note: Bold *p*-values indicate statistically significant results (*p* < 0.05).

## Data Availability

The data that support the findings of this study are available on request from the corresponding author. The data are not publicly available due to privacy or ethical restrictions.
